# Gut microbiota mediates cognitive impairment in young mice after multiple neonatal exposures to sevoflurane

**DOI:** 10.18632/aging.203193

**Published:** 2021-06-28

**Authors:** Meiyu Liu, Shaoyong Song, Qingcai Chen, Jianhong Sun, Wei Chu, Yunzeng Zhang, Fuhai Ji

**Affiliations:** 1 Department of Anesthesiology, First Affiliated Hospital of Soochow University, Suzhou, Jiangsu, China; 2 Department of Anesthesiology, The Affiliated Hospital of Yangzhou University, Yangzhou, Jiangsu, China; 3 Medical School of Soochow University, Suzhou, Jiangsu, China; 4 Joint International Research Laboratory of Agriculture and Agri-Product Safety, Ministry of Education of China, Yangzhou University, Yangzhou, Jiangsu, China

**Keywords:** anesthesia, sevoflurane, gut microbiota, cognitive impairment

## Abstract

Multiple exposures to anesthesia may increase the risk of cognitive impairment in young children. However, the mechanisms underlying this neurodevelopmental disorder remain elusive. In this study, we investigated alteration of the gut microbiota after multiple neonatal exposures to the anesthetic sevoflurane and the potential role of microbiota alteration on cognitive impairment using a young mice model. Multiple neonatal sevoflurane exposures resulted in obvious cognitive impairment symptoms and altered gut microbiota composition. Fecal transplantation experiments confirmed that alteration of the microbiota was responsible for the cognitive disorders in young mice. Microbiota profiling analysis identified microbial taxa that showed consistent differential abundance before and after fecal microbiota transplantation. Several of the differentially abundant taxa are associated with memory and/or health of the host, such as species of Streptococcus, Lachnospiraceae, and Pseudoflavonifractor. The results reveal that abnormal composition of the gut microbiota is a risk factor for cognitive impairment in young mice after multiple neonatal exposures to sevoflurane and provide insight into a potential therapeutic strategy for sevoflurane-related neurotoxicity.

## INTRODUCTION

Young children who experienced multiple surgical procedures under general anesthesia before 3 years of age are likely to fall within a high-risk category as defined by the recent US Food and Drug Administration warning: “repeated anesthesia in surgery or lengthy use of sedatives and general anesthetics (> 3 hours) may affect the brain development in children < 3 years” [1–4]. However, single and short-duration anesthesia and surgery do not cause a detectable impact on neurodevelopment in children [5, 6]. Remarkably, a recent prospective study revealed that children receiving multiple exposures to anesthesia show problems in behaviors, executive function, and reading [7]. Thus, exposure to anesthetics during childhood may lead to long-term deficits in neurodevelopmental function of children [8–10].

Sevoflurane, which has the favorable properties of low pungency, rapid onset, rapid offset, and low blood/gas ratio, is the most commonly used inhalational anesthetic agent for pediatric patients [11, 12]. Numerous preclinical studies have shown that repeated sevoflurane exposure during the neonatal period causes cognitive impairment and neurotoxicity in young rodents and monkeys [13–17], which may be associated with nerve cell apoptosis [18], impairment of synaptic development [19], neurogenesis inhibition [20], or impairment of glial cells development [21]. In addition, treatments and possible mechanisms for cognitive impairment caused by repeated sevoflurane exposure have been extensively studied in the developing central nervous system. However, the underlying mechanisms of peripheral responses on cognitive impairment in young mice after multiple neonatal exposures to sevoflurane remain elusive.

The gut microbiota is a peripheral microorganism community in the digestive tract that helps to maintain dynamic metabolic ecological balance [22, 23]. The gut microbiota, not only through the nervous system (the gut–brain neuroanatomical pathway) but also through the endocrine system, immune system, and metabolic system, affords significant advantages to the host throughout development [24]. However, abnormal composition of the gut microbiota can substantially influence the function and microenvironment of the brain [25–28], through the three routes of the gut–brain axis (immune, neuroendocrine, and vagal nerve pathway) [28–30]. Mounting evidence indicates that inhalation anesthesia can alter the composition of the gut microbiota in mice [31]. Recent studies have revealed significant interactions between alteration of the gut microbiota and cognitive behavior [32, 33]. However, scant attention has been paid to investigating the relationship between cognitive impairment and abnormal composition of the gut microbiota in young mice after multiple neonatal exposures to sevoflurane.

Therefore, we used a mouse model of multiple neonatal exposures to sevoflurane to evaluate the effects of anesthesia on cognitive function. Sequencing of the 16S ribosomal RNA gene and analysis of fecal samples were used to assess changes in the gut microbiota. In addition, we examined the effects of fecal microbiota transplantation on cognitive behavior in antibiotic-induced pseudo germ-free mice. The primary objective was to determine whether multiple neonatal exposures to sevoflurane impact on the gut microbiota of the young mice to induce cognitive impairment. The findings will contribute to an improved understanding of the mechanisms of anesthesia-related neurotoxicity.

## RESULTS

### Comparison of cognitive behavior between anesthesia group and control group

**Figure 1:**
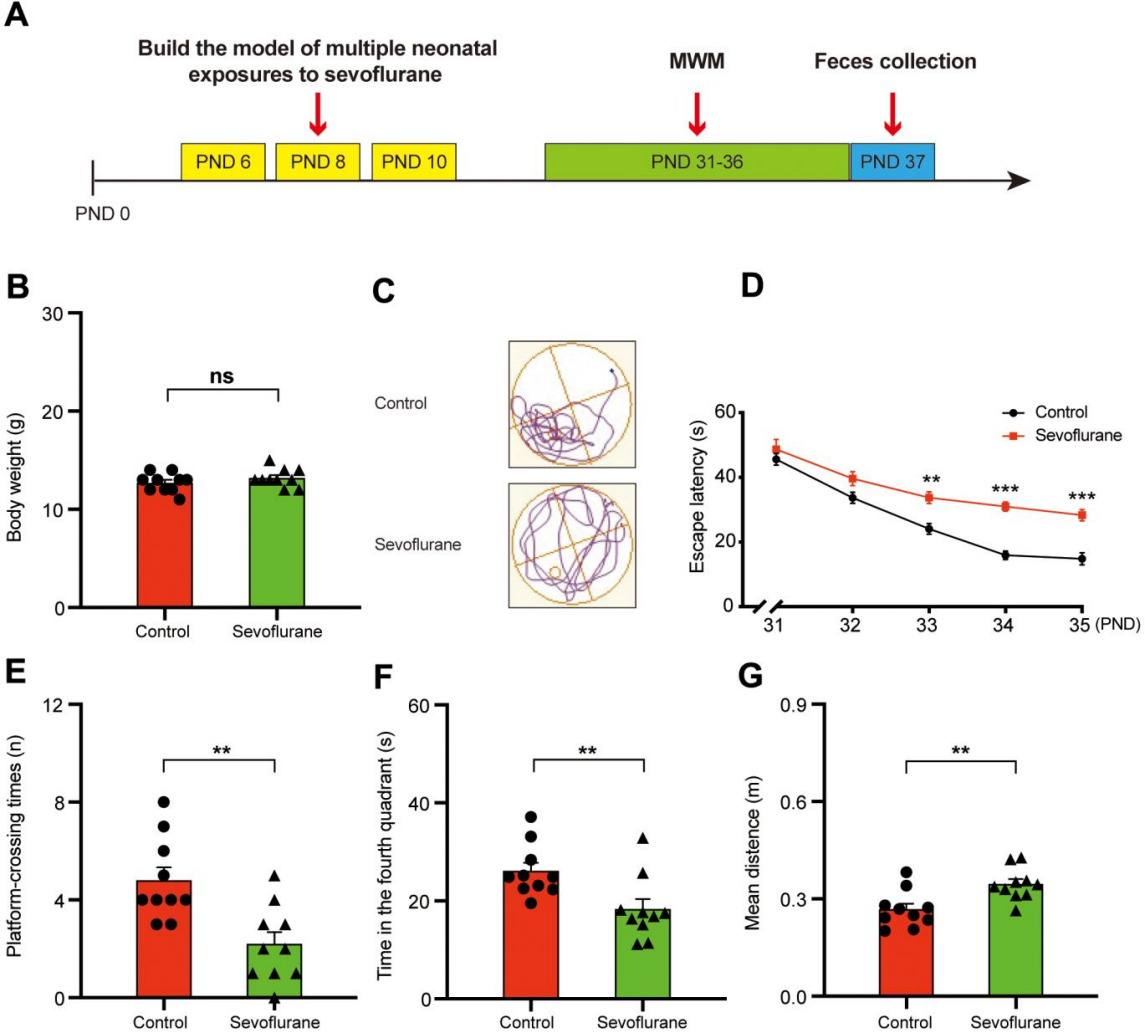
**Morris water maze test for control and sevoflurane-treated mice.** (**A**) Experimental schedule: 3% sevoflurane was applied for 2 h daily on PND 6, 8, and 10, MWM test on PND 31–36, and fecal sample collection for 16S ribosomal RNA gene sequencing and fecal bacteria transplant on PND 37. (**B**) Body weight (Student’s *t*-test, *p* > 0.05). (**C**) Trace plot of control and sevoflurane-treated mice in the MWM test. (**D**) Escape latency (two-way ANOVA; Time: *F*_4,72_ = 67.43, *p* < 0.001; Group: *F*_1,18_ = 43.14, *p* < 0.001; Interaction: *F*_4,72_ = 3.857, *p* = 0.007). (**E**) Platform-crossing instances (Student’s *t*-test, *p* = 0.0021). (**F**) Time spent in the fourth quadrant (Student’s *t*-test, *p* = 0.0092). (**G**) Mean distance from the platform (Student’s *t*-test, *p* = 0.0041). PND: postnatal day; ANOVA: analysis of variance; MWM: Morris water maze. Data are shown as mean ± SEM (*n* = 10). Significance: * *p* < 0.05, ** *p* < 0.01, *** *p* < 0.001, ns: non-significant.

The study design is summarized in Figure 1A. The mice from the anesthesia group (i.e., receiving 3% sevoflurane three times on PND (postnatal day) 6, 8, and 10 (for 2 h at each application) exhibited longer escape latency in the MWM (Morris water maze) test during the training phase on PND 33–35 (Figure 1D). In addition, the anesthesia group exhibited reduced spatial positioning capability as demonstrated by the results from the probe trial on PND 36, including showing fewer platform-crossing instances (4.80 ± 0.53 vs 2.20 ± 0.49, p = 0.0021; Figure 1E), less time spent in the fourth quadrant (26.10 ± 1.70 s vs 18.31 ± 2.06 s, p = 0.0092; Figure 1F), and longer mean distance from the platform (0.27 ± 0.02 m vs 0.35 ± 0.02 m, p = 0.0041; Figure 1G) compared with those of the control group. These results suggested that the sevoflurane treatment may result in defective memory and cognitive function of the treated mice [19, 34].

### Alterations in the gut microbiota composition between control group and anesthesia group

Recent studies have demonstrated that certain microorganisms in the gut microbiota play critical roles in the memory and cognitive behaviors of the hosts [30, 35]. We sought to determine whether the cognitive behavioral difference between the control and sevoflurane-treated mice was associated with a change in the gut microbiota. Given that body weight has a strong impact on the gut microbiota [36], we first measured the body weight of the control and sevoflurane-treated mice. No difference in body weight was observed between the two groups (12.70 ± 0.30 g vs 13.20 ± 0.29 g, p = 0.25; Figure 1B).

**Figure 2:**
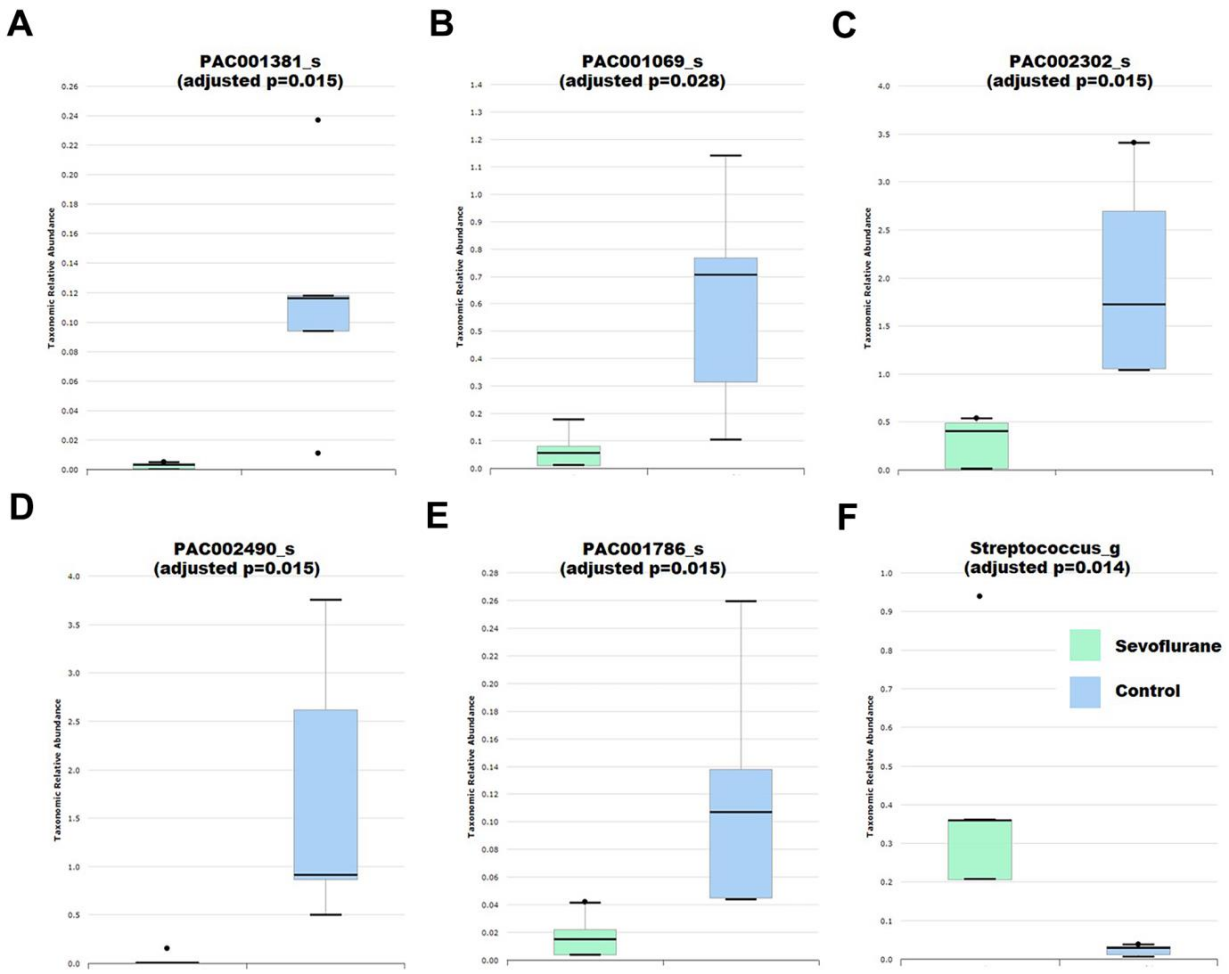
**Differential abundance of gut bacteria between control (*n* = 5) and anesthesia (*n* = 4) mice.** (**A**) Species PAC001381_s. (**B**) Species PAC001069_s. (**C**) Species PAC002302_s. (**D**) Species PAC002490_s. (**E**) Species PAC001786_s. (**F**) Genus *Streptococcus*. Taxa were assigned based on the PKSSU4.0 database implemented in the EzBioCloud platform.

Fecal samples were then collected for 16S ribosomal RNA gene sequencing. Based on the comprehensive PKSSU4.0 database implemented in the EzBioCloud platform, the bacterial taxonomic composition of the control and sevoflurane-treated mice was determined and compared. No significant difference in alpha-diversity between the two groups was observed, as indicated by the Shannon and Simpson indices (p > 0.05). Principal coordinate analysis indicated that the bacterial communities of the anesthesia group were not significantly different from those of the control group. These results suggested that sevoflurane treatment did not significantly alter the overall taxonomic composition of the gut microbiota. Previous reports also suggested that cognitive dysfunction caused by streptozotocin-induced diabetes or high-cholesterol diet did not significantly alter the gut microbiota composition in mice [37, 38]. However, we observed that multiple taxa exhibited significant differences in relative abundance between the sevoflurane-treated and control mice (Supplementary Table 1 and Figure 2). Among these taxa, several have been reported to be associated with memory and/or health of the hosts. For instance, the genus Streptococcus, which is an important pathogen causing neural damage and a risk factor for cerebral microbleeds and cognitive impairment [39, 40], exhibited increased relative abundance in the sevoflurane group. In contrast, the species PAC001381_s, an uncultured taxon belonging to the Lachnospiraceae, exhibited decreased relative abundance in sevoflurane-treated mice. Lachnospiraceae is an important member of the gut microbiota that can benefit the host in multiple aspects by producing beneficial metabolites, such as propionate and tryptophan [41]. Decreased Lachnospiraceae abundance in the gut microbiota were associated with a worse clinical profile, including higher frequencies of cognitive impairment [42]; while higher Lachnospiraceae abundance were associated with good cognition behaviors independent of clinical variables [43]. Decreased relative abundance of two species affiliated with Pseudoflavonifractor, PAC002302_s and PAC002490_s, in the sevoflurane-treated mice was observed. Pseudoflavonifractor is positively associated with weight loss of obese patients and may benefit the host by producing short-chain fatty acids [44, 45]. The results suggested that the sevoflurane treatment showed a long-term impact on the gut microbiota, at least 27 days post the sevoflurane treatment as demonstrated by the microbiota profiling results.

### Effects of control group and anesthesia group gut microbiota transplant on MWM behavior in antibiotic-induced pseudo germ-free mice

**Figure 3:**
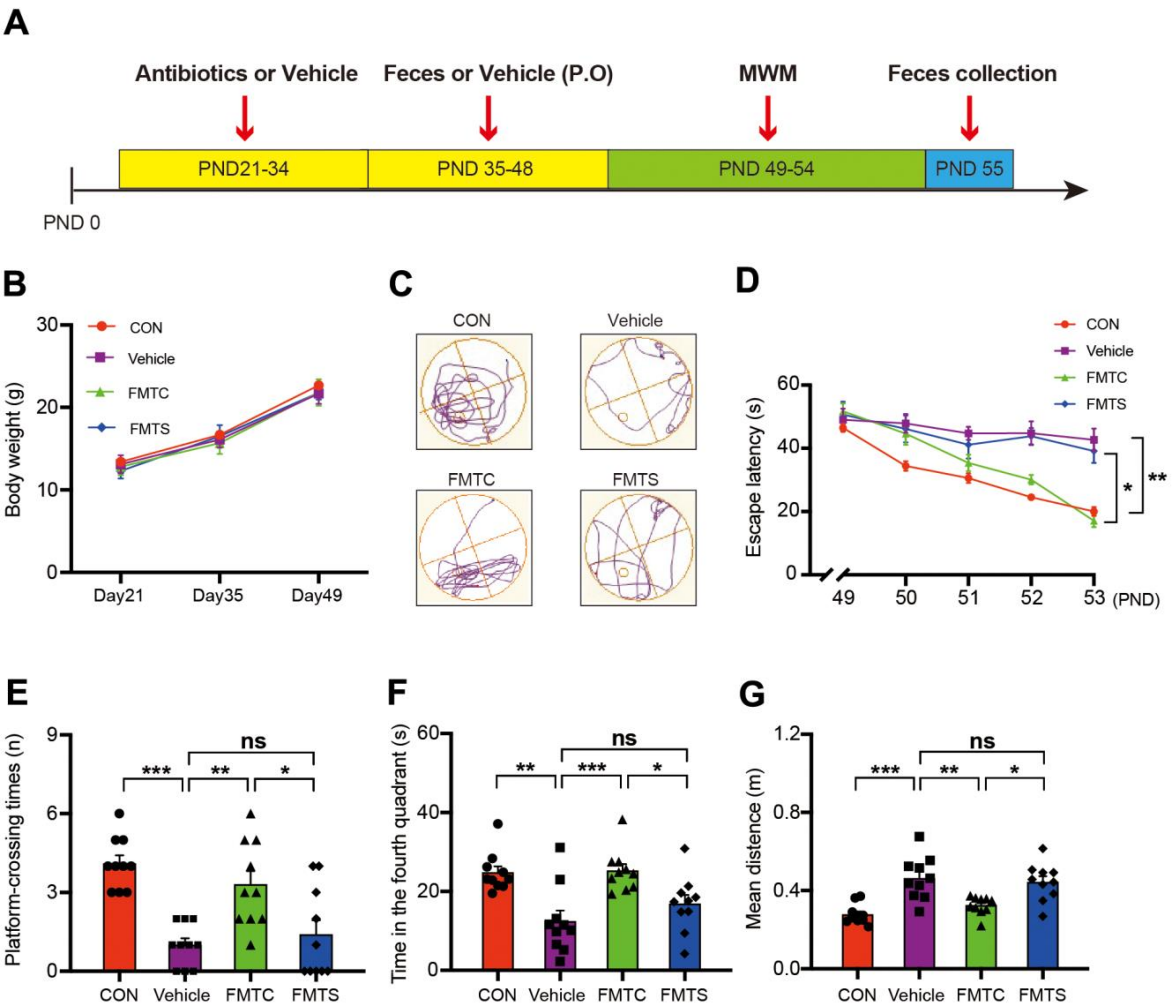
**Effects of transplantation of fecal microbiota from control and sevoflurane-treated mice on behavior of pseudo germ-free mice.** (**A**) Experimental summary: fecal microbiota transplantation effects on behavioral testing in pseudo germ-free mice. Wild-type male mice were first treated by administering high doses of antibiotic solution for 14 consecutive days on PND 21–34. Thereafter, mice were orally treated with fetal microbiota of control and anesthesia mice on PND 35–48. The MWM test was performed on PND 49–54. Fecal samples were collected for 16S ribosomal RNA gene sequencing testing on PND 55. (**B**) Body weight (two-way ANOVA; Time: *F*_2,72_ = 959.6, *p* < 0.001; Group: *F*_3,36_ = 1.795, *p* = 0.17; Interaction: *F*_6,72_ = 1.209, *p* = 0.31). (**C**) Trace plot of mice in the MWM test. (**D**) Escape latency (two-way ANOVA; Time: *F*_4,144_ = 35.46, *p* < 0.001; Group: *F*_3,36_ = 14.51, *p* < 0.001; Interaction: *F*_12,144_ = 4.436, *p* < 0.001). (**E**) Platform-crossing instances (one-way ANOVA; *F*_3,36_ = 12.20, *p* < 0.001). (**F**) Time spent in the fourth quadrant (one-way ANOVA; *F*_3,36_ = 8.812, *p* = 0.0002). (**G**) Mean distance from the platform (one-way ANOVA; *F*_3,36_ = 12.56, *p* < 0.001). PND: postnatal day; ANOVA: analysis of variance; MWM: Morris water maze. Data are shown as mean ± SEM (*n* = 10). * *p* < 0.05, ** *p* < 0.01, *** *p* < 0.001.

To examine whether the defective memory and cognitive function of the sevoflurane-treated mice were due to alteration of the gut microbiota composition, we performed microbiota transplantation experiments using the microbiota from the sevoflurane-treated mice and the control mice as donors and pseudo germ-free mice as recipients. The study design is summarized in Figure 3A. The pseudo germ-free mouse model was established by administering antibiotics at high doses for 14 consecutive days on PND 21–34. Gut microbiota from the control group and anesthesia group were transplanted into the gastrointestinal tract of pseudo germ-free mice for an additional 14 consecutive days on PND 35–48.

No obvious differences in body weight gain among the control (no treatment), pseudo germ-free mice (vehicle), pseudo germ-free mice with transplanted microbiota from the control mice used in the aforementioned experiment (FMTC), and pseudo germ-free mice with transplanted microbiota from the sevoflurane-treated mice used in the aforementioned experiment (FMTS) were observed (Figure 3B). Interestingly, the vehicle mice exhibited increased escape latency time during the training phase of the MWM test (16.31 ± 3.83 s vs 42.70 ± 11.25 s, p = 0.005 for PND 54; Figure 3D), and fewer platform-crossing instances (4.10 ± 0.31 vs 1.00 ± 0.26, p < 0.001; Figure 3E), less time spent in the fourth quadrant (24.80 ± 1.59 s vs 12.45 ± 2.71 s, p = 0.0011; Figure 3F), and longer mean distance from the platform (0.28 ± 0.02 m vs 0.46 ± 0.03 m, p < 0.001; Figure 3G) compared with those of the control group. The defective memory and cognitive function of the vehicle mice were recovered by regaining the regular full-spectrum microbiota (i.e., that of the FMTC mice); however, the microbiota derived from sevoflurane-treated mice (i.e., the FMTS mice) did not dramatically improve the cognitive function of the recipients as demonstrated by the MWM test (Figure 3D–3G).

### Effects of fecal microbiota transplant from anesthesia and control mice on the abundance of host gut microbiota

**Figure 4:**
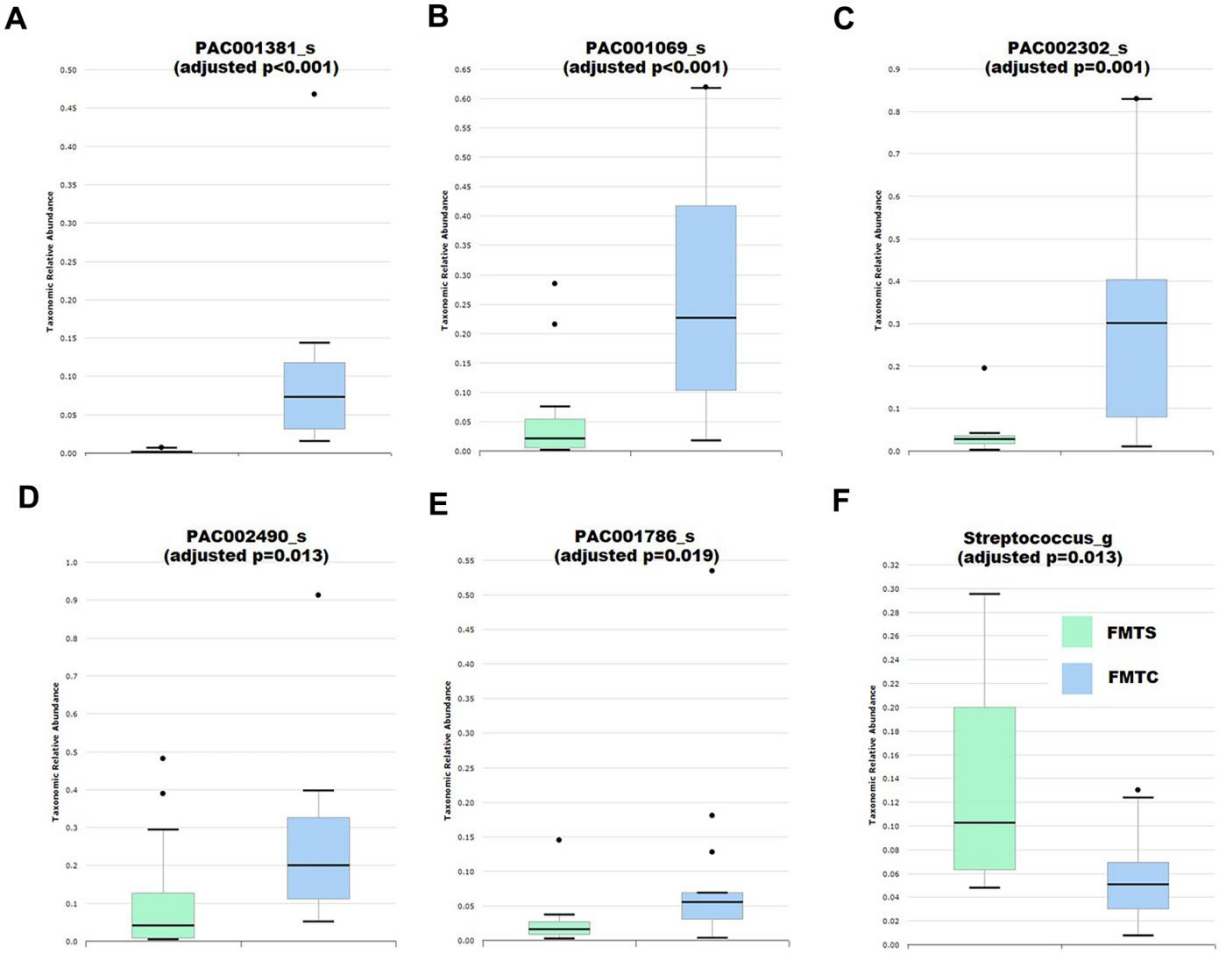
**Effects of fecal microbiota transplantation from anesthesia-treated (FMTS, *n* = 14) and control (FMTC, *n* = 13) mice on the composition of the host gut microbiota of pseudo-germ-free mice.** (**A**) Species PAC001381_s. (**B**) Species PAC001069_s. (**C**) Species PAC002302_s. (**D**) Species PAC002490_s. (**E**) Species PAC001786_s. (**F**) Genus *Streptococcus*. Taxa were assigned based on the PKSSU4.0 database implemented in the EzBioCloud platform.

Since all mice used for FMTS and FMTC treatments were on the same mental and gut microbiota composition background (pseudo germ-free vehicle mice), thus the cognitive behavior differences between the FMTS and FMTC mice were not due to the mental status or the original gut microbiota of the used mice, but were mainly due to the transplanted gut microbiota. Therefore, we performed the gut microbiota comparison analysis. The results demonstrated that the microbiota composition of the vehicle mice was very simple with majority of the members in the normal gut microbiota eliminated, while the microbiota composition of the FMTS and FMTC mice was dramatically different from that of the vehicle mice, suggesting the FMT experiments were successfully performed (Supplementary Figure 1). Following an identical bioinformatics analysis protocol, the microbiota composition of FMTS and FMTC mice was compared and the taxa differing significantly in abundance were identified (Supplementary Table 2 and Figure 4). A fraction of differentially abundant taxa identified in the FMTS vs FMTC experiment not overlapping with the abovementioned control vs sevoflurane experiment, which is frequently observed in fecal microbiota transplantation experiments [46]. However, it was interesting to note that several taxa showed consistency between the pre- and post-transplantation microbiota. Control or sevoflurane-treated fecal microbiota transplantation significantly improved or further aggravated the same changes in pseudo germ-free mice (Figure 4A–4F).

## DISCUSSION

The newborn period is an extremely important stage for brain development [47, 48]. For rodents, this critical neurodevelopmental process, also termed “the window of vulnerability”, is likely to occur during PND 7–30 when the neuronal architecture and brain function are dramatically trained and improved by environmental events, which may better equip an individual to cope with environmental challenges [49, 50]. Negative environmental factors experienced by an individual during this window of vulnerability can also lead to adverse reactions in brain development [51]. Consistent with the findings of previous studies [52, 53], the present results demonstrated that multiple neonatal exposures to sevoflurane induced cognitive impairment of the young host. However, the mechanisms underlying this anesthesia-triggered cognitive impairment remain elusive.

The present results demonstrated that those sevoflurane-treated neonatal mice that showed learning disability and memory impairment exhibited an altered gut microbiota composition compared with that of the control mice (Figure 2 and Supplementary Table 1). The fecal transplantation experiments using pseudo germ-free mice further demonstrated that the gut microbiota alternation is responsible for the learning disability and memory impairment of the host [54–56]. Comparison of microbiota profiles between the control and sevoflurane-treated mice (first experimental cycle) as well as the FMTC and FMTS mice (second experimental cycle) identified multiple taxa that exhibited consistent differential relative abundance (Figures 2, 4, Supplementary Tables 1, 2). Several of these taxa, such as Lachnospiraceae and *Streptococcus*, have been reported to be associated with learning and memory functions of the hosts [39, 40, 42, 43]. Accumulating evidence indicates that inhalational anesthetics can affect the process of microbial colonization [31, 57]. The current results further suggested that the memory and cognitive function impairment of the sevoflurane-treated host is due to, at least partially, altered relative abundance of certain crucial members of the gut microbiota.

Recent studies have demonstrated that the gut microbiota is an environmental factor that strongly impacts on brain development and behavior [23, 58, 59]. The gut microbiota is a relatively stable ecosystem; however, the composition and diversity of the gut microbiota is always in a dynamic state during the neonatal period [23]. Newborn babies’ intestines are rapidly colonized by an array of microbes from their mothers, which is characterized by low diversity; in addition, infants possess a stable gut microbial profile that is highly similar to the characteristic microbiota of an adult by the end of the third year of life [60–62]. Therefore, the first 3 years of life represent the most critical period for establishment of the gut microbiota to improve child growth and neurodevelopment, during which time the composition and diversity of the gut microbiota is readily affected by harmful environmental factors, such as bacterial infections and antibiotic treatment [63]. In this study, we found the pseudo germ-free mice exhibited cognitive impairment symptoms. Although it was hard to differentiate and quantify the roles of high dose antibiotics and the consequent gut microbiota alteration on the observed cognitive impairment in the pseudo germ-free mice [64], however, fecal microbiota transplanted from control mice, but not from sevoflurane-treated mice, reversed the detrimental effects on cognitive function in the pseudo germ-free mice, further demonstrating that the microbiota alternation triggered by sevoflurane treatment was responsible for the cognitive impairment in the sevoflurane-treated mice. Taken together, these findings show that multiple neonatal exposures to sevoflurane may strongly impact on microbial colonization of the gut, resulting in altered cognitive phenotypes in adulthood.

Several aspects were not addressed by the present study. First, we primarily focused on the microbial taxa that showed a consistent trend before and after fecal microbiota transplantation to investigate the mechanism of cognitive impairment, but other members of the gut microbiota might also be associated with cognitive function. Second, the microbial alpha-diversity failed to show an apparent difference in response to sevoflurane treatment. Although alpha-diversity is used as a measure of the diversity and richness of the unique microbial taxa within a sample, it should be emphasized that alpha-diversity may not be the sole criterion for gut microbiota dysbiosis [65]. Furthermore, some instances of gut microbiota dysbiosis show no changes in alpha-diversity [66]. Third, the germ-free mouse construct provides an optimal means of assessing long-term effects of specific bacteria on cognitive impairment, but the behavioral assessment of these mice is restricted to sterile isolator units to maintain the germ-free environment. Thus, pseudo germ-free mice provide a more amenable and cost-effective model for behavioral assessment. Finally, under the present experimental design, we observed elements of the gut microbiota that may participate in regulation of cognitive functions in young mice after multiple neonatal exposures to sevoflurane, further investigation is needed to elucidate the roles and the mechanisms of these bacteria on the cognitive functions, and how the microbiota alternation affects the brain tissue to cause the cognitive impairment in the sevoflurane-treated mice.

To the best of our knowledge, the present study is the first to show the effects of an altered gut microbiota on cognitive impairment in young mice after multiple neonatal exposures to sevoflurane. These findings indicate that neonatal exposures to sevoflurane may lead to abnormal composition of the gut microbiota, which is a potential risk factor for neurological development. With its novel insight into sevoflurane-related neurotoxicity in the developing brain, this study presents a promising foundation for further research on microbial manipulation to improve the safety of anesthesia care in children.

## MATERIALS AND METHODS

### Animals and anesthesia

The animal studies (ethical protocol number: 201910A204) were conducted in accordance with the guidelines and regulations of the Institutional Animal Care and Use Committee of Soochow University (Suzhou, China). C57BL/6J mice were purchased from the Shanghai Laboratory Animal Center (Shanghai, China) and housed in a specific pathogen-free animal room supplied with standard rodent food and water. The male pups were used in this study. The neonatal mice were randomly assigned to either of the two study groups (control or sevoflurane treatment) with the aid of a computer-generated table.

The animal model was described in our previous study [52]. The mice in the sevoflurane group received 3% sevoflurane with 60% oxygen (balanced with nitrogen) for three non-consecutive days (postnatal days [PND] 6, 8, and 10), for 2 h at each application (2 L/min fresh gas from the start up to 3 min for induction, followed by 1 L/min for maintenance) in a chamber using a Datex-Ohmeda anesthesia system (Madison, WI, USA), which conceptually mimics the multiple exposures of anesthesia in patients [67]. The control group received 60% oxygen in nitrogen for 2 h with an equal rate of flow in a chamber that was identical to the anesthesia chamber [13]. The sevoflurane concentration was continuously monitored using a gas analyzer (Vamos; Dräger Medical, Lübeck, Germany) during the anesthesia. The rectal temperature of the mice was maintained at 37 ± 0.5° C. After anesthesia, the mice were returned to home cages (metabolic cage: allowing feces to leak out to prevent eating each other's feces) under standard care [53, 68]. Given that previous studies demonstrated that anesthesia with 3% sevoflurane did not significantly change the pH, partial pressures of oxygen, partial pressures of carbon dioxide, and hematocrit of the young mice [52], we did not perform blood gas analysis of the mice in this study.

### Morris water maze (MWM) Test

A Morris water maze (MWM) test was conducted as previously described [69]. The water maze device, i.e., a round steel pool (150 cm diameter and 60 cm height) with a 10 cm diameter-size platform located in the center, was surrounded by a black curtain and located in an isolated, quiet room. The device was filled with water to a level 1.0 cm above the surface of the platform. Throughout the experiment, the water temperature was maintained at 22° C. The mice were trained to reach the platform for five consecutive days (PND 31–35) with four trials per day. In the training phase each mouse was placed in the water and given 60 s to locate the platform; if the mice could not find the platform within 60 s, they were gently guided to the platform and allowed to remain there for 15 s. The time and routine for each mouse to reach the platform was recorded by video-tracking software (ANY-maze, Stoelting, CO, USA) to evaluate its spatial learning ability. The platform was removed in the testing phase on PND 36, then a 60 s probe trial was performed for assessment of memory function. The number of times the mice crossed the platform area and time spent in the fourth quadrant were recorded. The mice were warmed and dried with a heat lamp after each test.

### Pseudo germ-free mice model establishment

Pseudo germ-free mice were established as described by previous studies [51, 70] with slight modification. Briefly, C57BL/6J male mice were treated with a broad-spectrum antibiotics cocktail (ampicillin 1 g/L, neomycin sulfate 1 g/L, and metronidazole 1 g/L; Sigma-Aldrich, St Louis, MO, USA) in drinking water for 14 consecutive days. The drinking solution was renewed every 2 days.

### Fecal microbiota transplant and MWM test

The male mice were placed in a clean cage after the MWM test. The 30 male mice were divided into the sevoflurane group (15) and control group (15). Ten mice in each group were prepared for fecal microbiota transplantation and the remaining mice in each group were used for 16S rDNA sequencing. The mice were killed on day 37 and the feces samples were collected from the ileocecal region and placed in a sterilized centrifuge tube. Feces were stored in a −80° C freezer until analysis and transplantation [71]. The fecal microbiota was prepared by diluting 1 g of the fecal sample obtained from the anesthesia group or control group mice in 10 mL sterile PBS. The fecal material was suspended and 0.2 mL of the suspension was guided by gavage into each mouse recipient for 14 consecutive days, and the pseudo germ-free mice were used as the recipients [70]. The MWM test was performed for the control (i.e., untreated), vehicle mice (i.e., pseudo germ-free), FMTC (i.e., pseudo germ-free mice with transplanted microbiota from the control mice used in the aforementioned experiment), and FMTS (i.e., pseudo germ-free mice with transplanted microbiota from the sevoflurane-treated mice used in the aforementioned experiment) as described in section 2.2.

### High-throughput 16S rDNA sequencing of the fecal samples and bioinformatics analyses

The fecal samples from the control and sevoflurane-treated mice used in the first cycle experiment, as well as the FMTC, and FMTS and vehicle mice used in the second cycle, were collected immediately from the ileocecal region after the MWM test (Figures 1A, 3A). Samples were placed in 1.5 ml tubes, snap-frozen on dry ice, and stored at −80° C. The 16S rDNA high-throughput sequencing was performed by Sangon Biotech Co., Ltd (Shanghai, China). DNA was extracted using EZNA Soil DNA Kits (Omega, Doraville, GA, USA). The 16S rDNA V3–V5 region was amplified with the primer set 338F (5′-ACTCCTACGGGAGGCAGC-3′) and 806R (5′-GGACTACHVGGGTWTCTAAT-3′). Triplicate PCR reactions for each sample were performed and merged for sequencing. The reverse primer contained a sample barcode and both primers were connected with an Illumina sequencing adapter. The PCR products were purified and sequenced on an Illumina MiSeq PE300 platform. Original sequencing data from the sample were sorted by unique barcodes, followed by removal of the barcode, linker, and PCR primer sequences. The resultant paired-end sequencing data were merged using FLASH [72], and the merged fastq files were analyzed using the 16S rRNA gene-based Microbiome Taxonomic Profiling pipeline implemented on the EzBioCloud server (https://www.ezbiocloud.net/) with the EzBioCloud 16S rDNA database version PKSSU4.0 employed [73]. Comparisons of the taxonomic composition between the control and the sevoflurane-treated mice (named as Sevoflurane herein) or the FMTC and FMTS mice using the comparative MTP analyzer implemented in EzBioCloud, and the alpha- and beta-diversity between the control and Sevoflurane groups or FMTC and FMTS groups, were conducted using normalized data with variation in the gene copy number considered. The taxonomic biomarkers for each group were identified using the linear discriminant analysis effect size (LEfSe) algorithm [74] using the aforementioned normalized data; the taxa with an adjusted p-value (false discovery rate) < 0.05 were considered to be significantly group-associated biomarkers.

The sequencing data have been deposited in the CNGB CNSA database under the Bioproject accession no. CNP0001462.

### Statistical analysis

Values presented are expressed as the mean ± SEM. Statistical analyses were performed using SPSS version 17.0 software (SPSS Inc., Chicago, IL, USA). Escape path length and escape latency in the MWM test were analyzed using two-way analysis of variance (ANOVA). Other data were analyzed using one-way ANOVA followed by *post-hoc* Tukey’s test, Student’s *t*-test, or Fisher’s exact test. *P*-values less than 0.05 were considered statistically significant.

## Supplementary Material

Supplementary Figure 1

Supplementary Table 1

Supplementary Table 2
