# Supplementary material for: Gut microbiota mediates cognitive impairment in young mice after multiple neonatal exposures to sevoflurane
Source: Aging (Albany NY). 2021 Jun 28;13(12):16733–48. doi: 10.18632/aging.203193 (PMC8266337; doi:10.18632/aging.203193)
Supplement: Supplementary Figure 1 [file aging-13-203193-s001.pdf]

SUPPLEMENTARY FIGURE

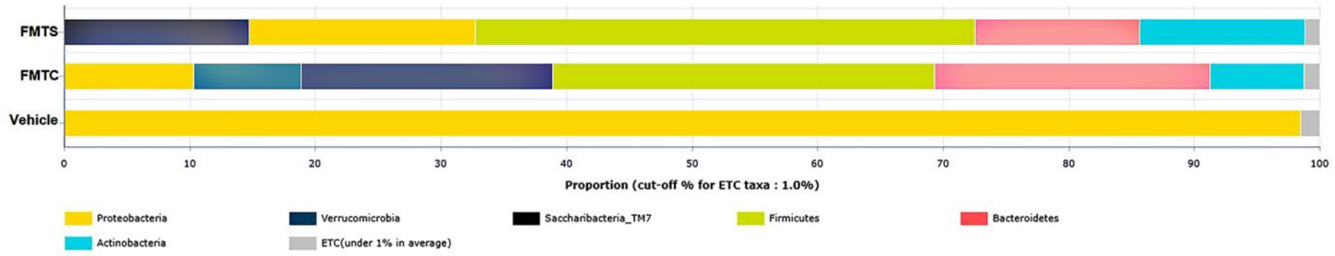

Supplementary Figure 1. Stacked histogram showing the relative abundance of bacterial community in the vehicle (pseudo germ-free), FMTC and FMTS mice. The taxa were shown at phylum level.
